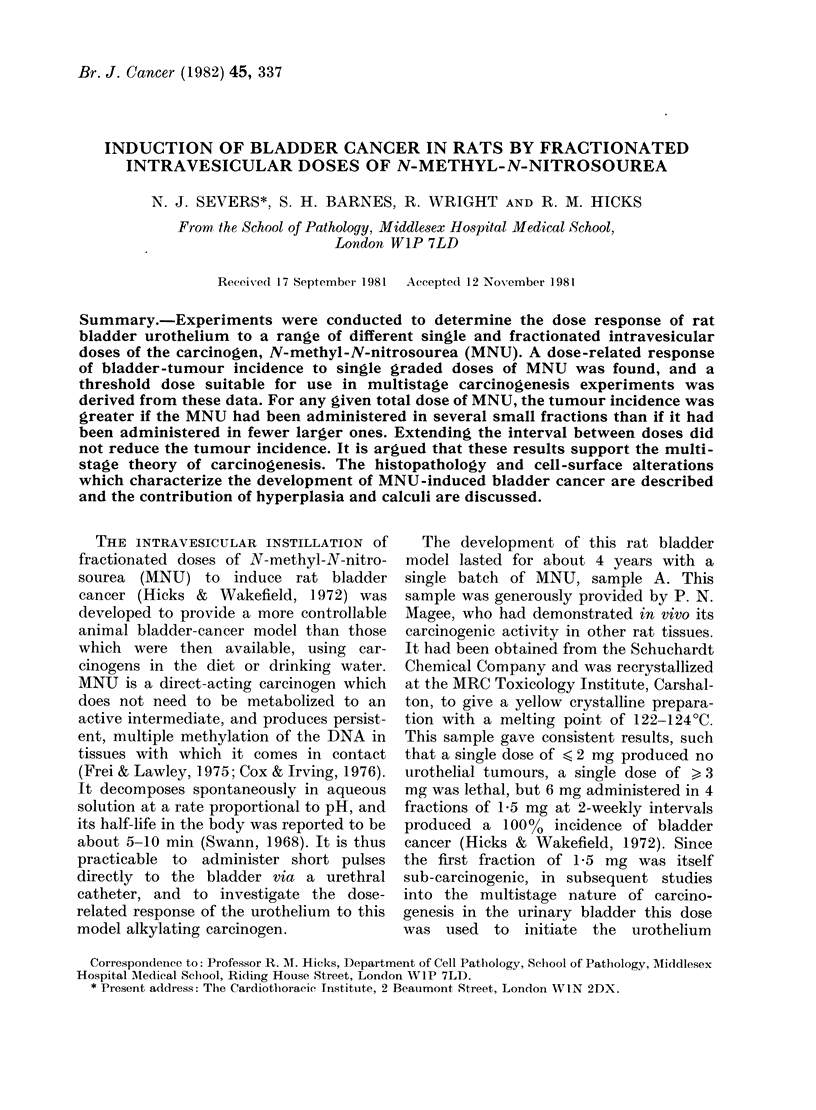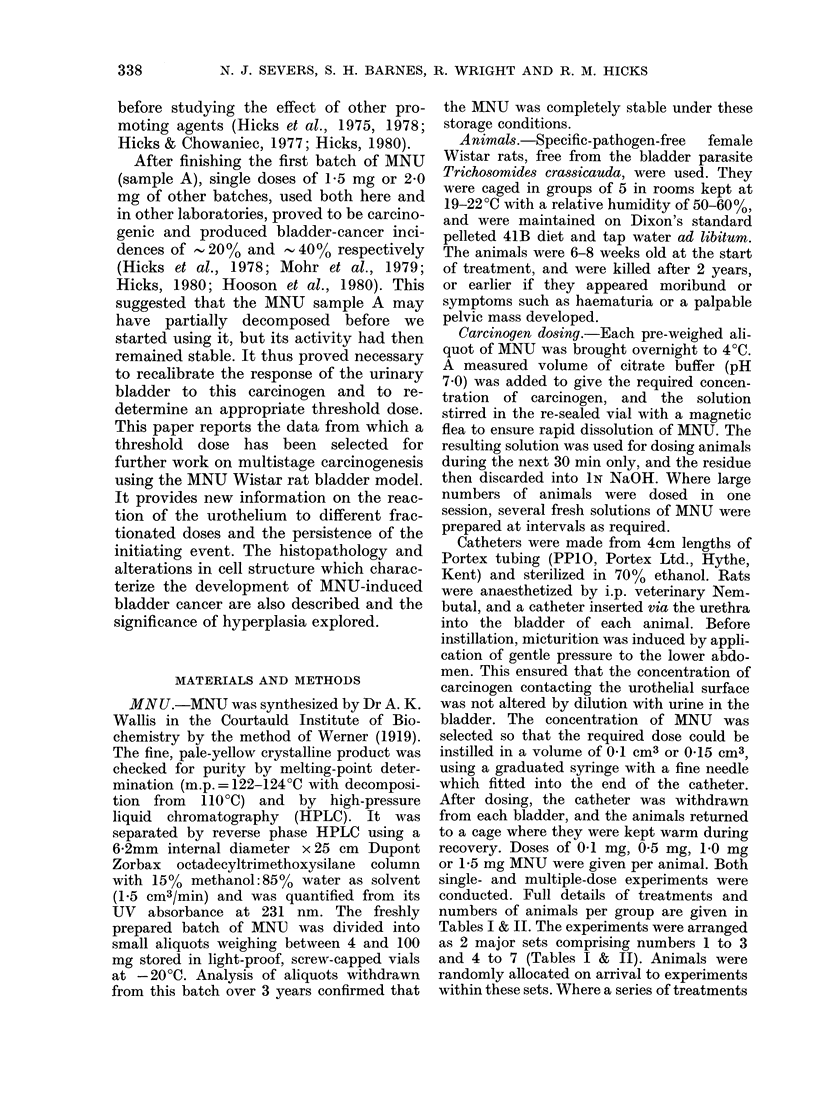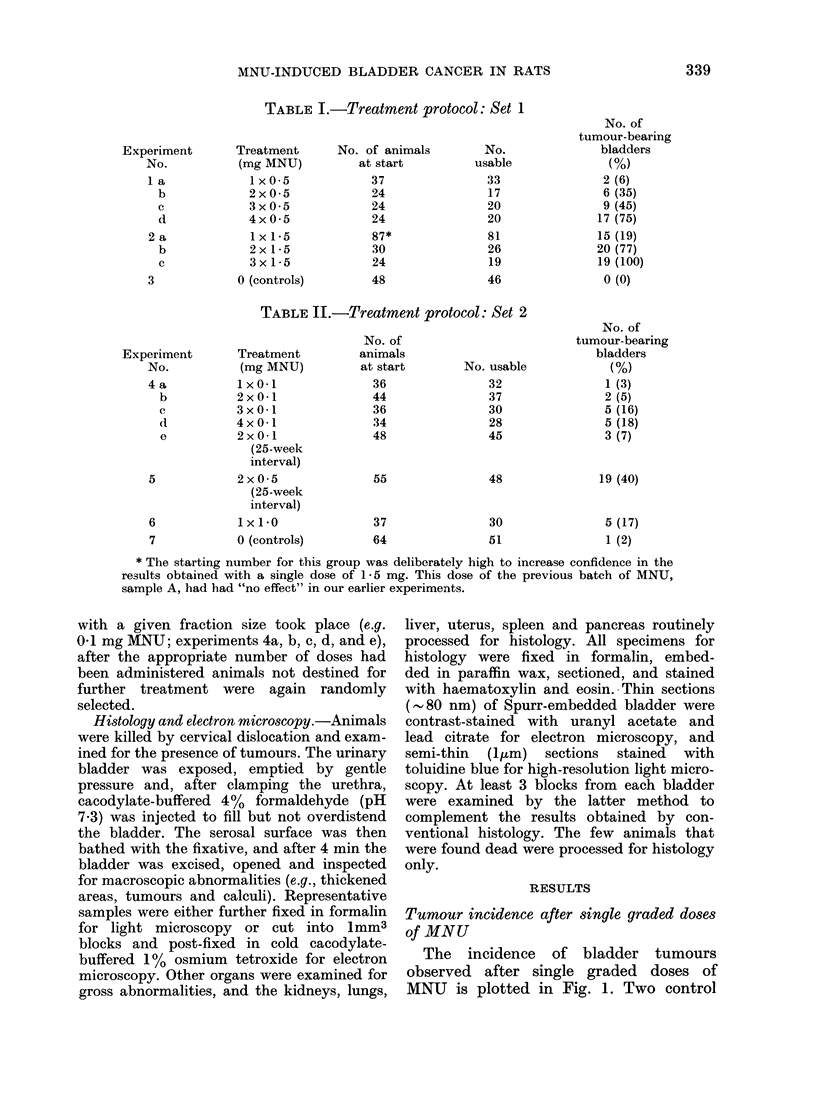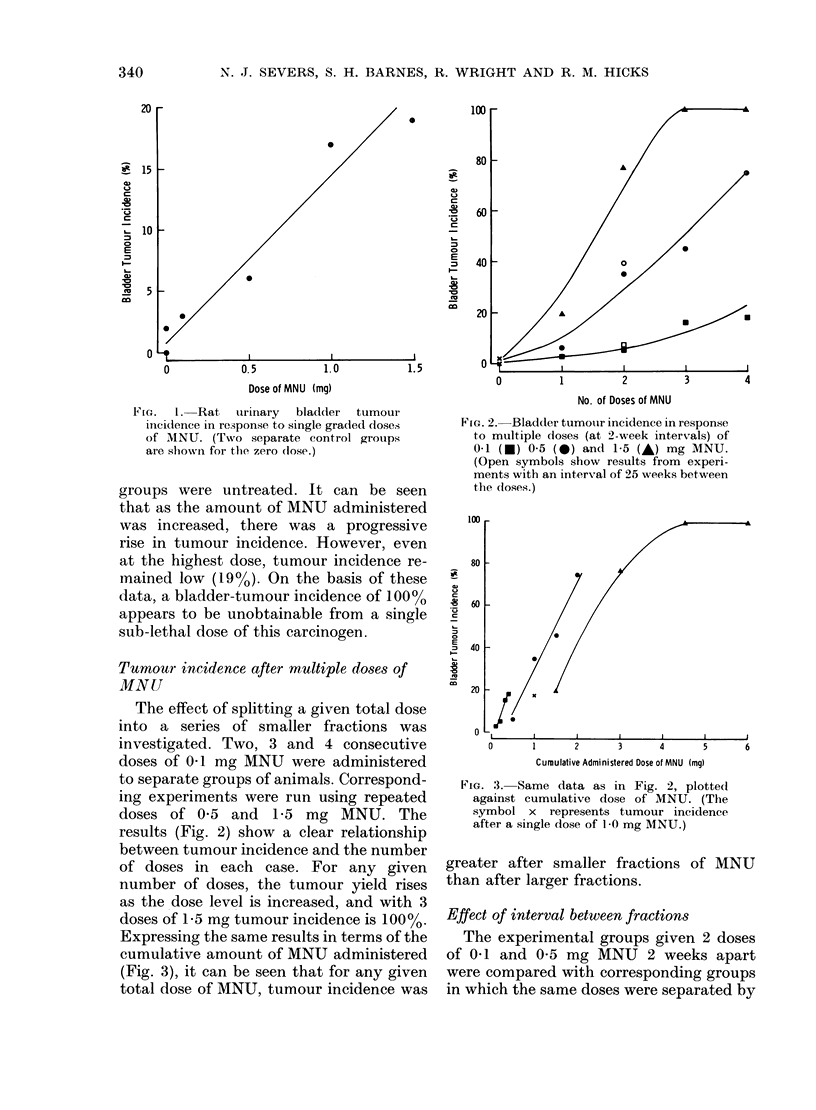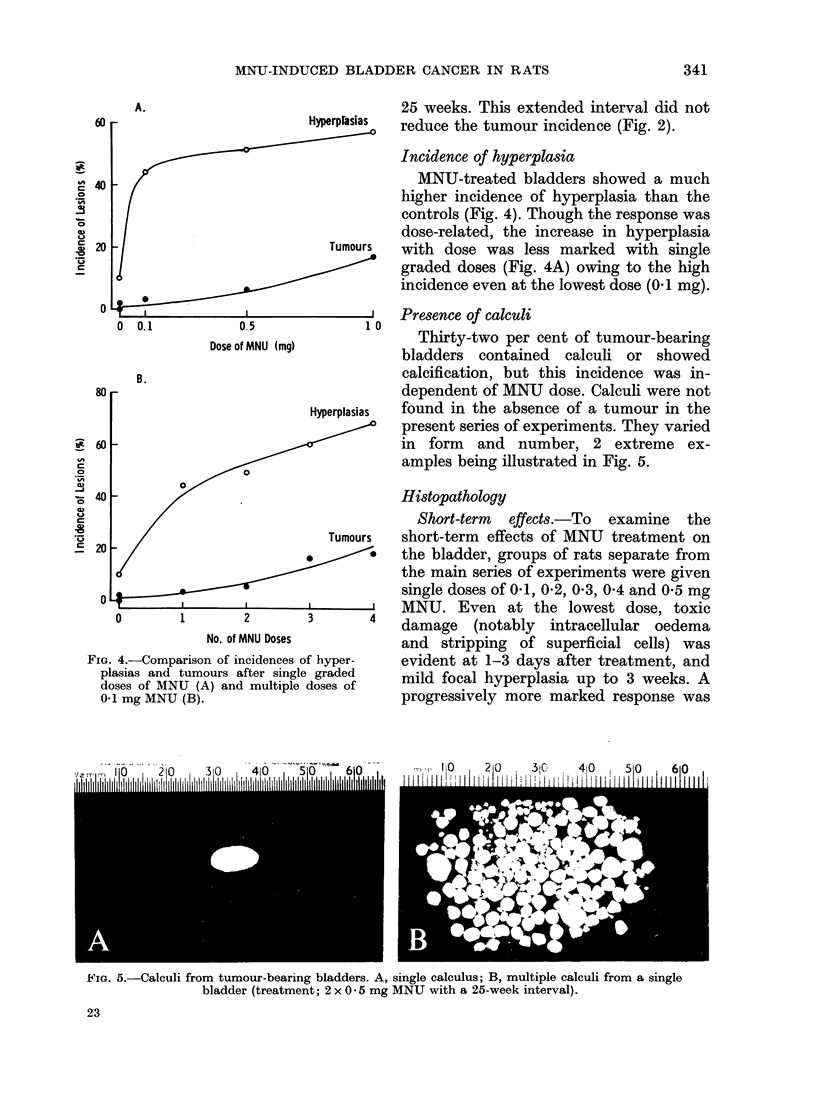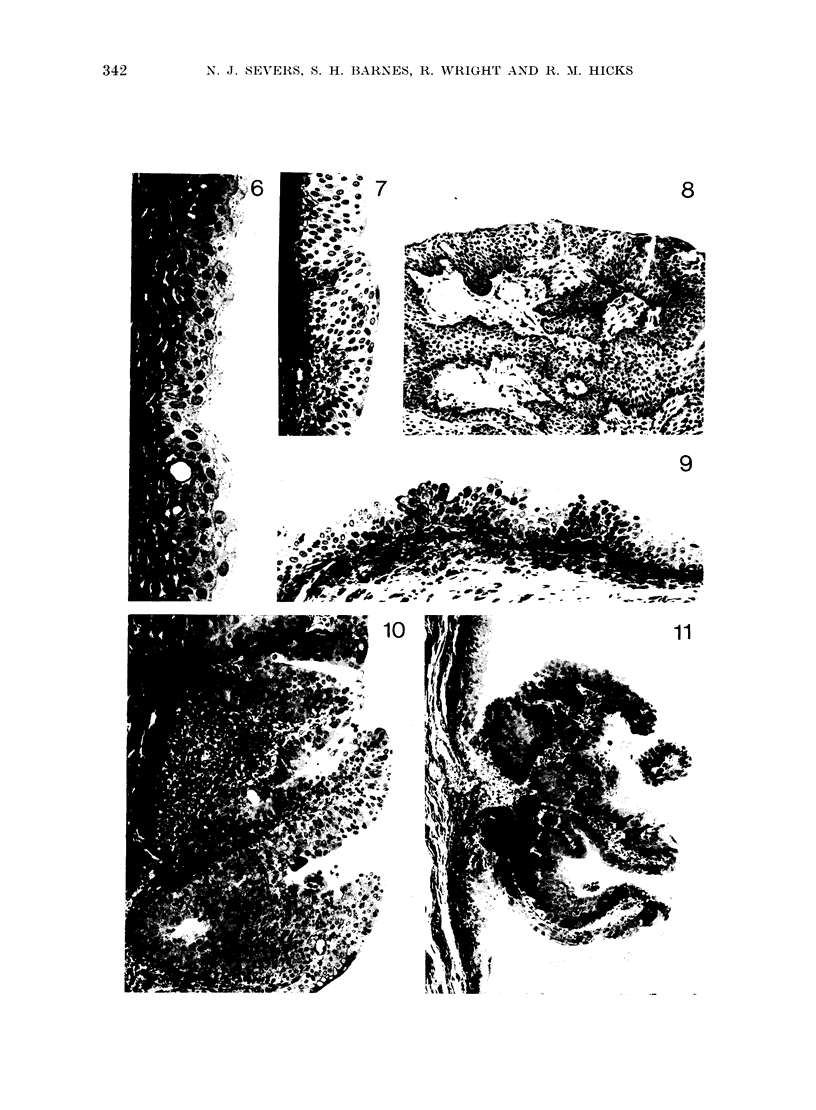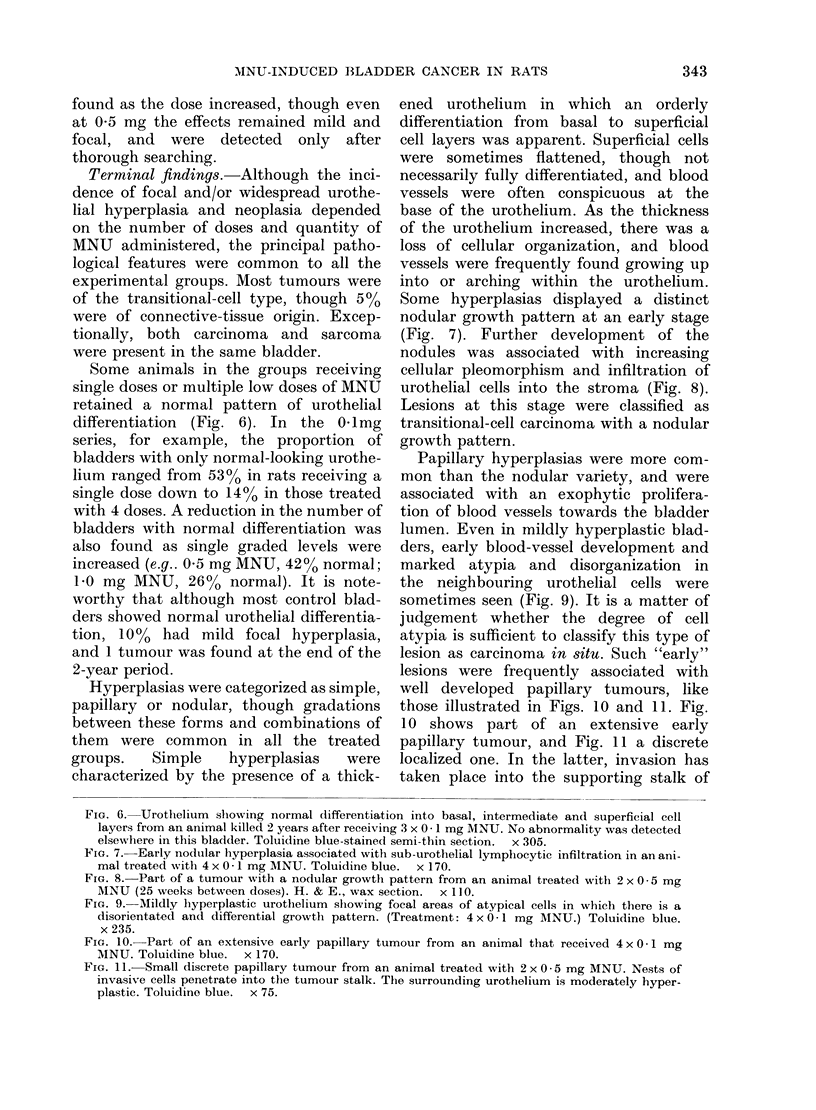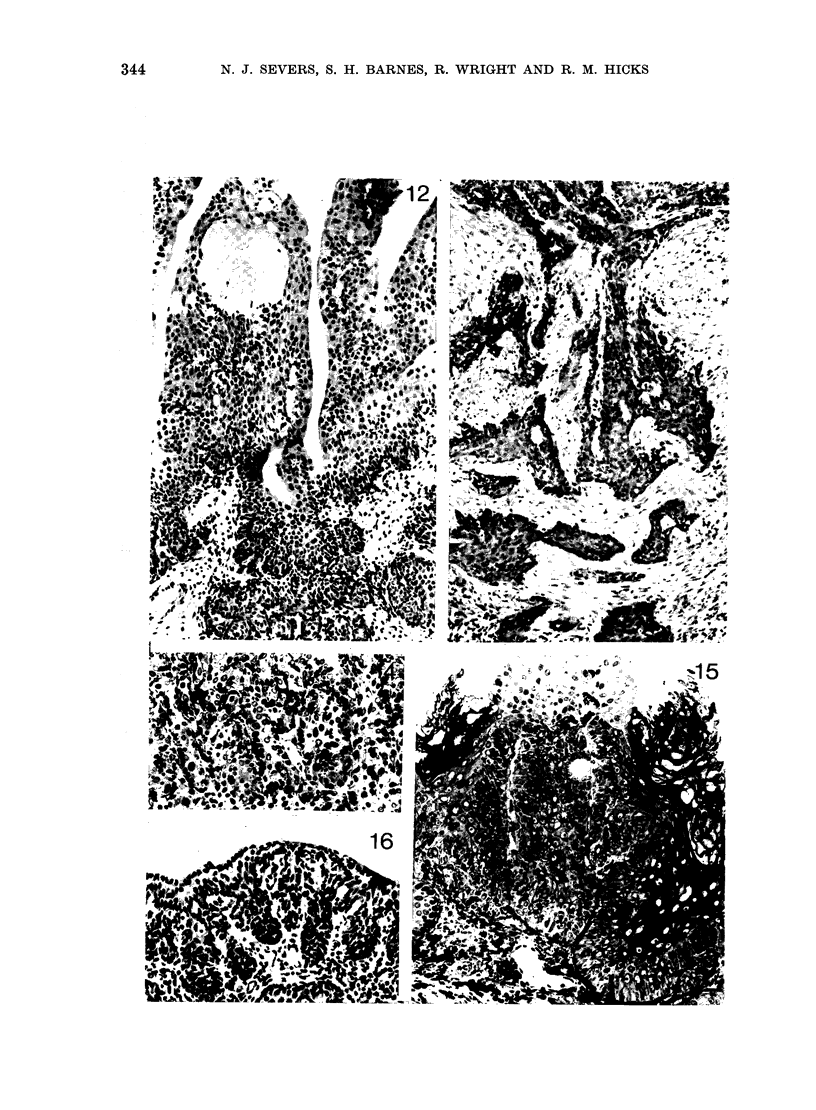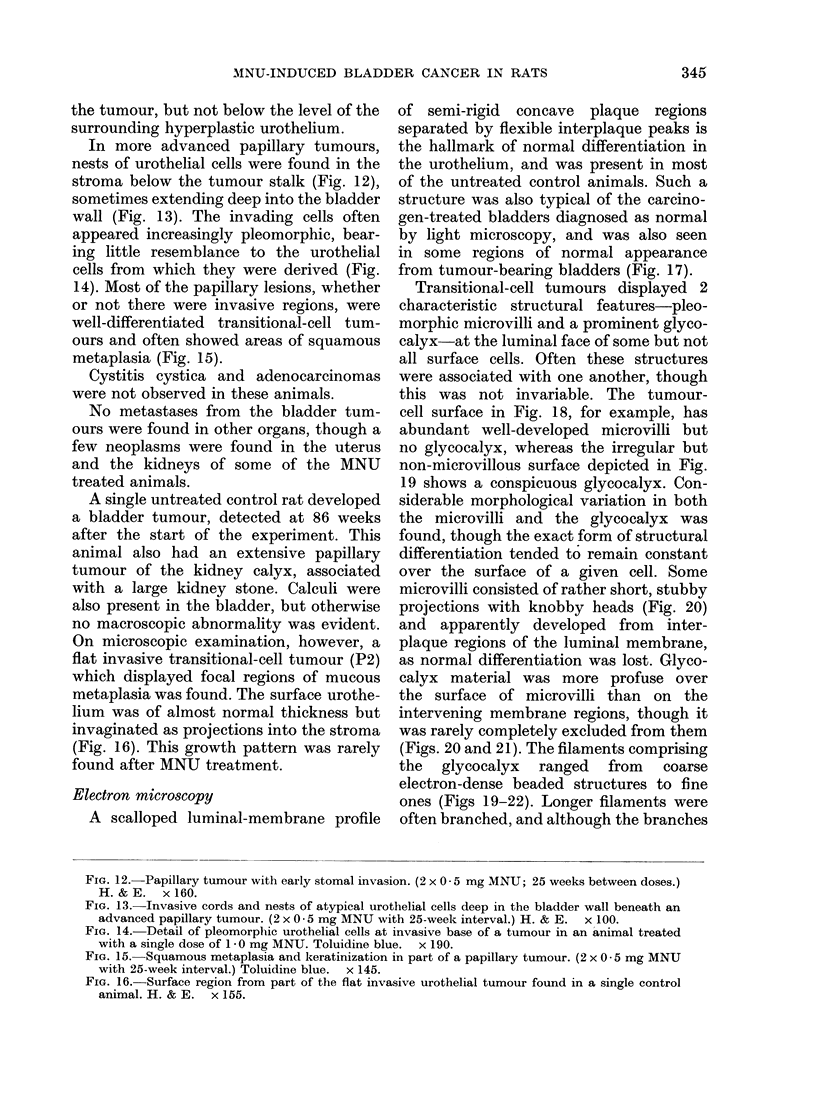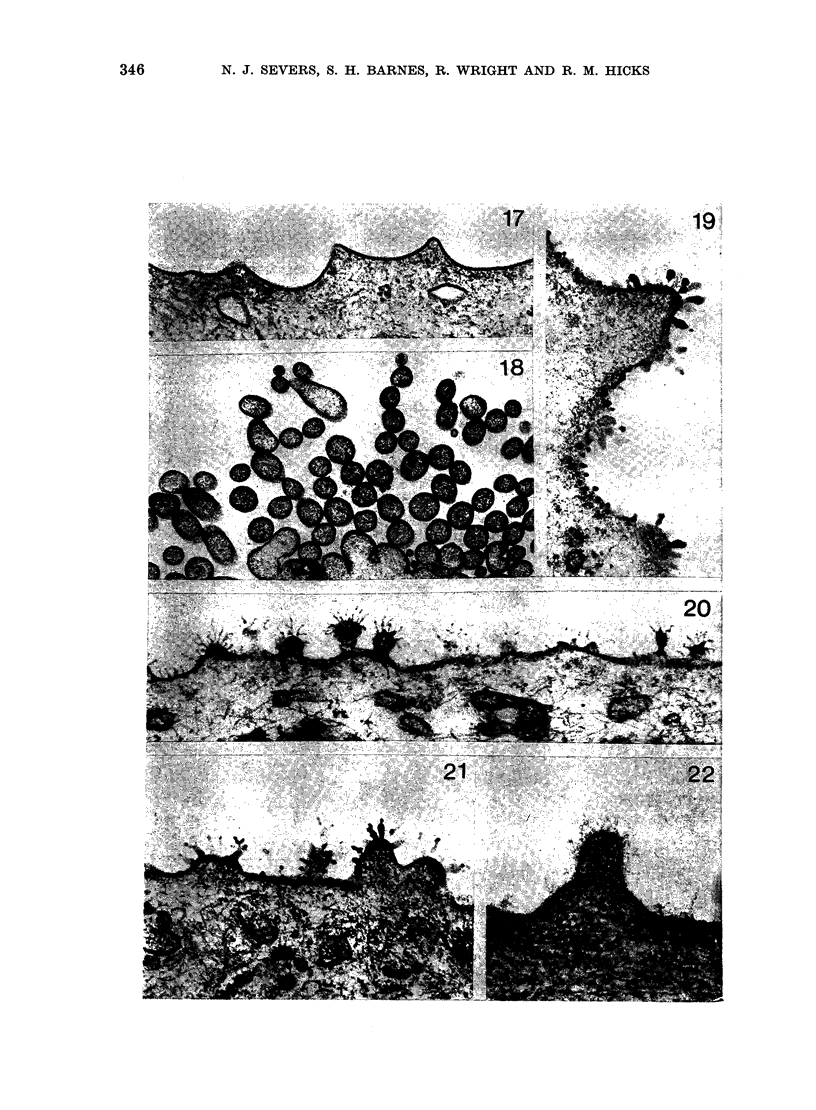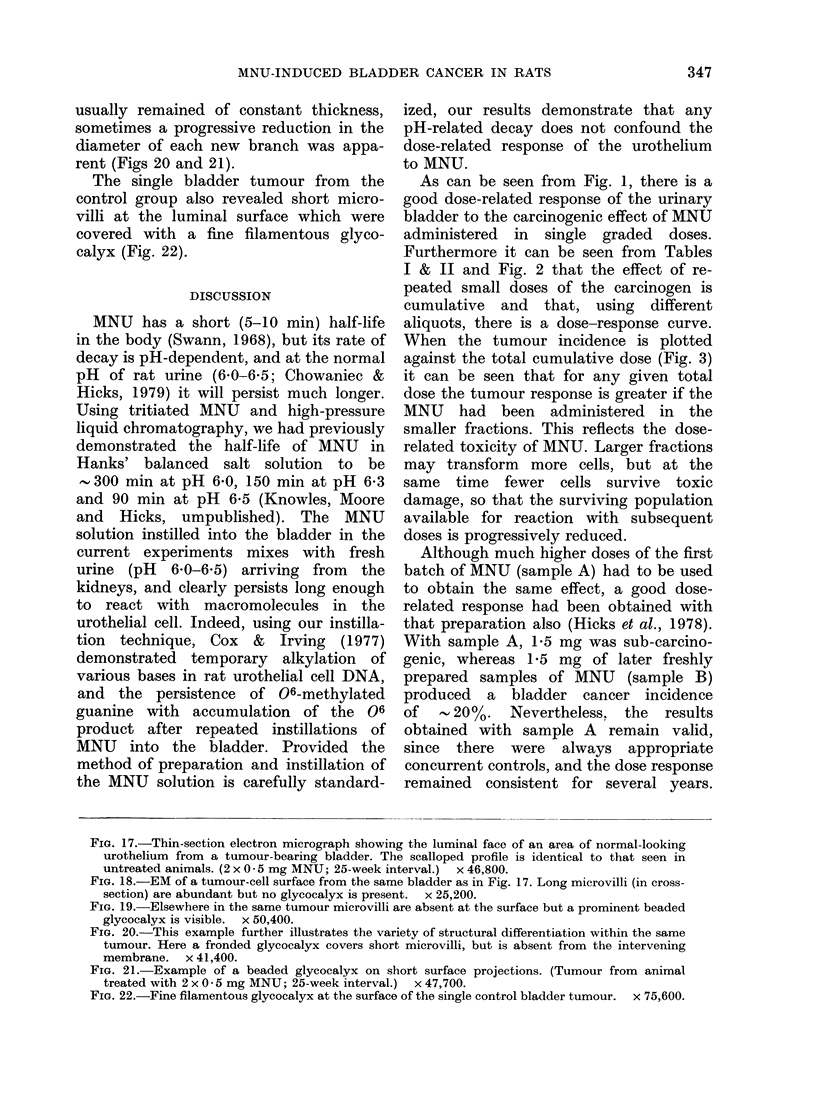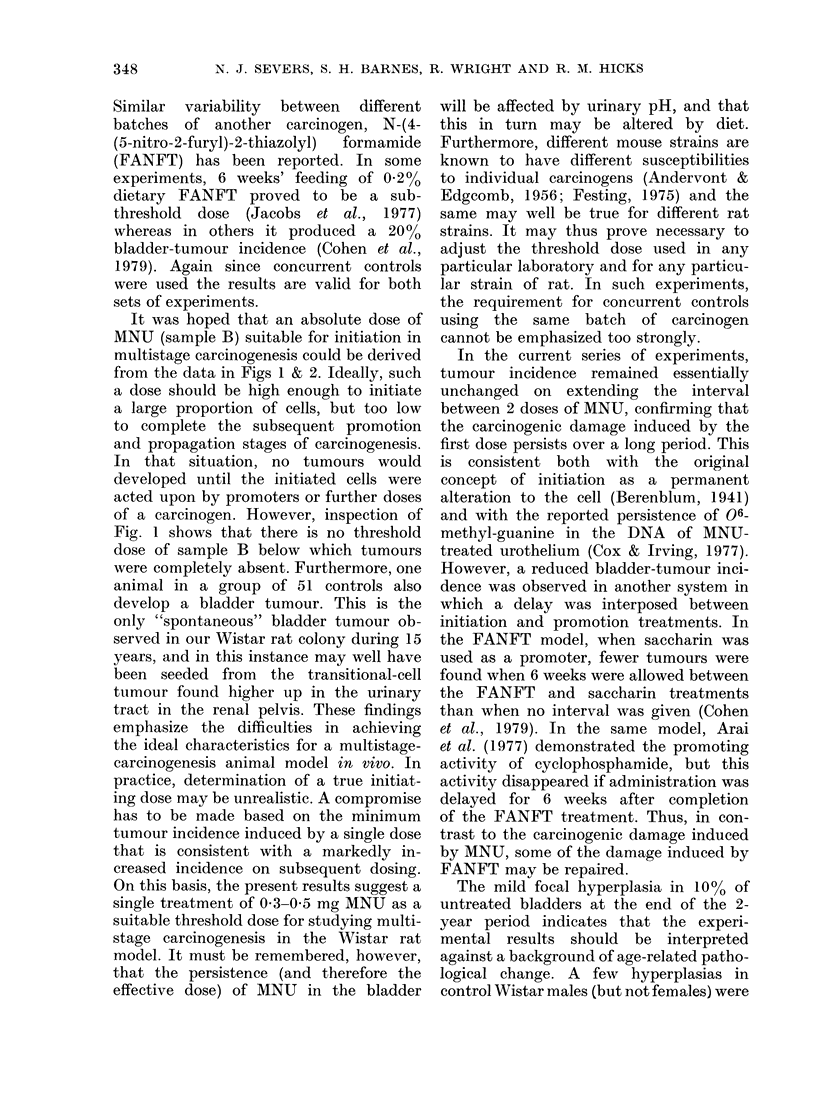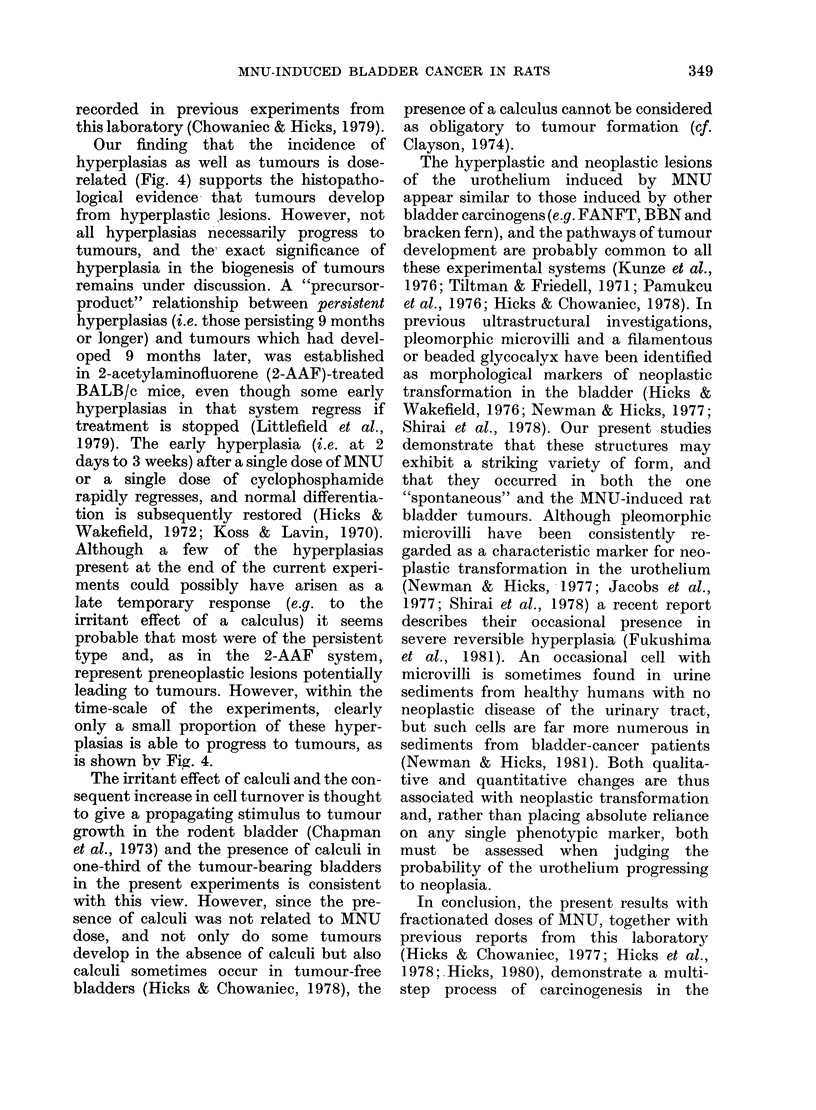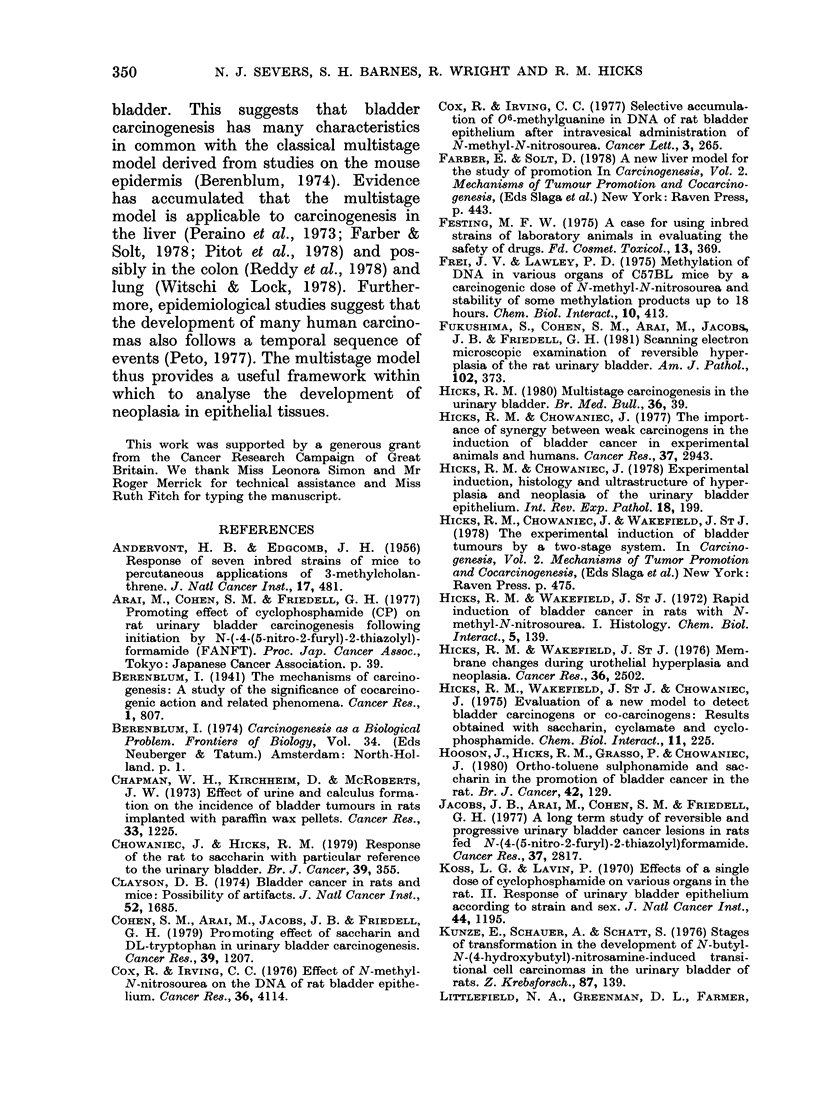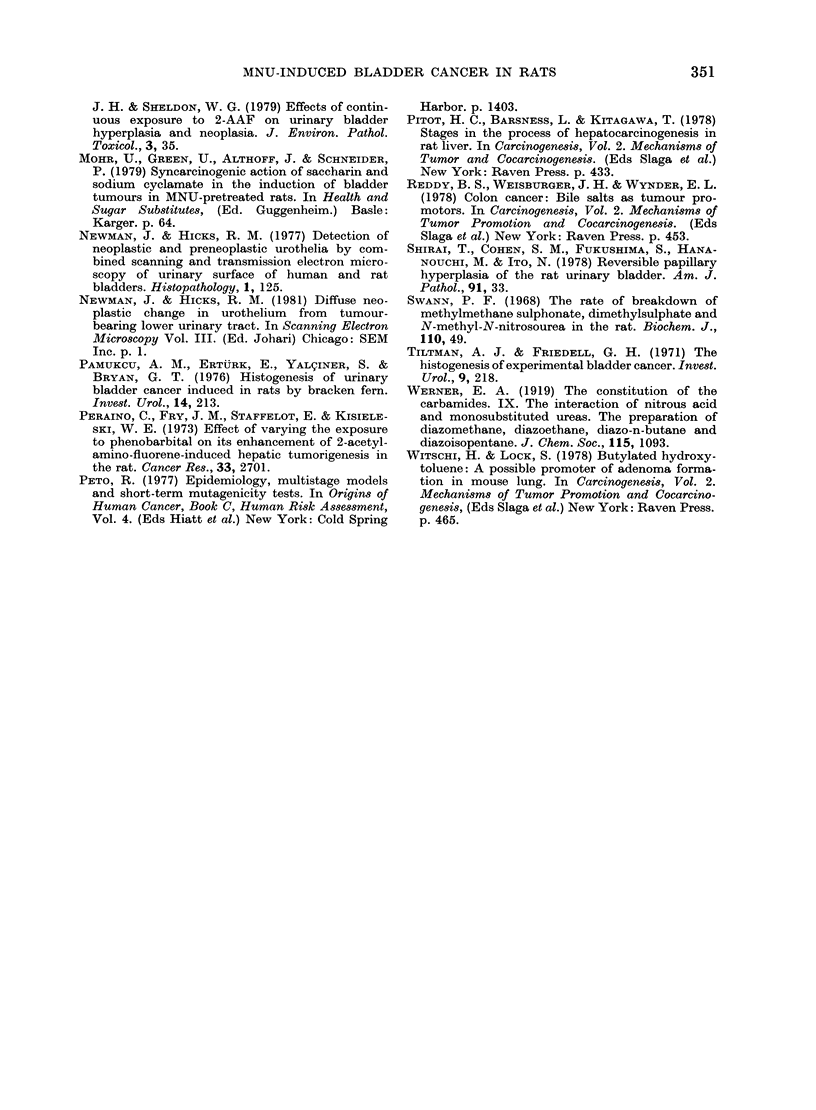# Induction of bladder cancer in rats by fractionated intravesicular doses of N-methyl-N-nitrosourea.

**DOI:** 10.1038/bjc.1982.60

**Published:** 1982-03

**Authors:** N. J. Severs, S. H. Barnes, R. Wright, R. M. Hicks

## Abstract

**Images:**


*Br. J. Cancer* (1982) **45**, 337

### INDUCTION OF BLADDER CANCER IN RATS BY FRACTIONATED INTRAVESICULAR DOSES OF *N*-METHYL-*N*-NITROSOUREA

N. J. SEVERS*, S. H. BARNES, R. WRIGHT AND R. M. HICKS

*From the School of Pathology, Middlesex Hospital Medical School, London W1P 7LD*

Received 17 September 1981 Accepted 12 November 1981

**Summary.—Experiments were conducted to determine the dose response of rat bladder urothelium to a range of different single and fractionated intravesicular doses of the carcinogen, *N*-methyl-*N*-nitrosourea (MNU). A dose-related response of bladder-tumour incidence to single graded doses of MNU was found, and a threshold dose suitable for use in multistage carcinogenesis experiments was derived from these data. For any given total dose of MNU, the tumour incidence was greater if the MNU had been administered in several small fractions than if it had been administered in fewer larger ones. Extending the interval between doses did not reduce the tumour incidence. It is argued that these results support the multistage theory of carcinogenesis. The histopathology and cell-surface alterations which characterize the development of MNU-induced bladder cancer are described and the contribution of hyperplasia and calculi are discussed.**

THE INTRAVESICULAR INSTILLATION of fractionated doses of *N*-methyl-*N*-nitrosourea (MNU) to induce rat bladder cancer (Hicks & Wakefield, 1972) was developed to provide a more controllable animal bladder-cancer model than those which were then available, using carcinogens in the diet or drinking water. MNU is a direct-acting carcinogen which does not need to be metabolized to an active intermediate, and produces persistent, multiple methylation of the DNA in tissues with which it comes in contact (Frei & Lawley, 1975; Cox & Irving, 1976). It decomposes spontaneously in aqueous solution at a rate proportional to pH, and its half-life in the body was reported to be about 5–10 min (Swann, 1968). It is thus practicable to administer short pulses directly to the bladder *via* a urethral catheter, and to investigate the dose-related response of the urothelium to this model alkylating carcinogen.

The development of this rat bladder model lasted for about 4 years with a single batch of MNU, sample A. This sample was generously provided by P. N. Magee, who had demonstrated *in vivo* its carcinogenic activity in other rat tissues. It had been obtained from the Schuchardt Chemical Company and was recrystallized at the MRC Toxicology Institute, Carshalton, to give a yellow crystalline preparation with a melting point of 122–124°C. This sample gave consistent results, such that a single dose of $\leq 2$ mg produced no urothelial tumours, a single dose of $\geq 3$ mg was lethal, but 6 mg administered in 4 fractions of 1·5 mg at 2-weekly intervals produced a 100% incidence of bladder cancer (Hicks & Wakefield, 1972). Since the first fraction of 1·5 mg was itself sub-carcinogenic, in subsequent studies into the multistage nature of carcinogenesis in the urinary bladder this dose was used to initiate the urothelium

Correspondence to: Professor R. M. Hicks, Department of Cell Pathology, School of Pathology, Middlesex Hospital Medical School, Riding House Street, London W1P 7LD.
* Present address: The Cardiothoracic Institute, 2 Beaumont Street, London W1N 2DX.

before studying the effect of other promoting agents (Hicks *et al.*, 1975, 1978; Hicks & Chowaniec, 1977; Hicks, 1980).

After finishing the first batch of MNU (sample A), single doses of 1·5 mg or 2·0 mg of other batches, used both here and in other laboratories, proved to be carcinogenic and produced bladder-cancer incidences of ~20% and ~40% respectively (Hicks *et al.*, 1978; Mohr *et al.*, 1979; Hicks, 1980; Hooson *et al.*, 1980). This suggested that the MNU sample A may have partially decomposed before we started using it, but its activity had then remained stable. It thus proved necessary to recalibrate the response of the urinary bladder to this carcinogen and to redetermine an appropriate threshold dose. This paper reports the data from which a threshold dose has been selected for further work on multistage carcinogenesis using the MNU Wistar rat bladder model. It provides new information on the reaction of the urothelium to different fractionated doses and the persistence of the initiating event. The histopathology and alterations in cell structure which characterize the development of MNU-induced bladder cancer are also described and the significance of hyperplasia explored.

### MATERIALS AND METHODS

*MNU.*—MNU was synthesized by Dr A. K. Wallis in the Courtauld Institute of Biochemistry by the method of Werner (1919). The fine, pale-yellow crystalline product was checked for purity by melting-point determination (m.p. = 122–124°C with decomposition from 110°C) and by high-pressure liquid chromatography (HPLC). It was separated by reverse phase HPLC using a 6·2mm internal diameter × 25 cm Dupont Zorbax octadecyltrimethoxysilane column with 15% methanol:85% water as solvent (1·5 $cm^3$/min) and was quantified from its UV absorbance at 231 nm. The freshly prepared batch of MNU was divided into small aliquots weighing between 4 and 100 mg stored in light-proof, screw-capped vials at −20°C. Analysis of aliquots withdrawn from this batch over 3 years confirmed that the MNU was completely stable under these storage conditions.

*Animals.*—Specific-pathogen-free female Wistar rats, free from the bladder parasite *Trichosomides crassicauda*, were used. They were caged in groups of 5 in rooms kept at 19–22°C with a relative humidity of 50–60%, and were maintained on Dixon's standard pelleted 41B diet and tap water *ad libitum*. The animals were 6–8 weeks old at the start of treatment, and were killed after 2 years, or earlier if they appeared moribund or symptoms such as haematuria or a palpable pelvic mass developed.

*Carcinogen dosing.*—Each pre-weighed aliquot of MNU was brought overnight to 4°C. A measured volume of citrate buffer (pH 7·0) was added to give the required concentration of carcinogen, and the solution stirred in the re-sealed vial with a magnetic flea to ensure rapid dissolution of MNU. The resulting solution was used for dosing animals during the next 30 min only, and the residue then discarded into 1N NaOH. Where large numbers of animals were dosed in one session, several fresh solutions of MNU were prepared at intervals as required.

Catheters were made from 4cm lengths of Portex tubing (PP1O, Portex Ltd., Hythe, Kent) and sterilized in 70% ethanol. Rats were anaesthetized by i.p. veterinary Nembutal, and a catheter inserted *via* the urethra into the bladder of each animal. Before instillation, micturition was induced by application of gentle pressure to the lower abdomen. This ensured that the concentration of carcinogen contacting the urothelial surface was not altered by dilution with urine in the bladder. The concentration of MNU was selected so that the required dose could be instilled in a volume of 0·1 $cm^3$ or 0·15 $cm^3$, using a graduated syringe with a fine needle which fitted into the end of the catheter. After dosing, the catheter was withdrawn from each bladder, and the animals returned to a cage where they were kept warm during recovery. Doses of 0·1 mg, 0·5 mg, 1·0 mg or 1·5 mg MNU were given per animal. Both single- and multiple-dose experiments were conducted. Full details of treatments and numbers of animals per group are given in Tables I & II. The experiments were arranged as 2 major sets comprising numbers 1 to 3 and 4 to 7 (Tables I & II). Animals were randomly allocated on arrival to experiments within these sets. Where a series of treatments

TABLE I.—*Treatment protocol: Set* 1

| Experiment No. | Treatment (mg MNU) | No. of animals at start | No. usable | No. of tumour-bearing bladders (%) |
|---|---|---|---|---|
| 1 a | 1 × 0·5 | 37 | 33 | 2 (6) |
| b | 2 × 0·5 | 24 | 17 | 6 (35) |
| c | 3 × 0·5 | 24 | 20 | 9 (45) |
| d | 4 × 0·5 | 24 | 20 | 17 (75) |
| 2 a | 1 × 1·5 | 87* | 81 | 15 (19) |
| b | 2 × 1·5 | 30 | 26 | 20 (77) |
| c | 3 × 1·5 | 24 | 19 | 19 (100) |
| 3 | 0 (controls) | 48 | 46 | 0 (0) |

TABLE II.—*Treatment protocol: Set* 2

| Experiment No. | Treatment (mg MNU) | No. of animals at start | No. usable | No. of tumour-bearing bladders (%) |
|---|---|---|---|---|
| 4 a | 1 × 0·1 | 36 | 32 | 1 (3) |
| b | 2 × 0·1 | 44 | 37 | 2 (5) |
| c | 3 × 0·1 | 36 | 30 | 5 (16) |
| d | 4 × 0·1 | 34 | 28 | 5 (18) |
| e | 2 × 0·1 (25-week interval) | 48 | 45 | 3 (7) |
| 5 | 2 × 0·5 (25-week interval) | 55 | 48 | 19 (40) |
| 6 | 1 × 1·0 | 37 | 30 | 5 (17) |
| 7 | 0 (controls) | 64 | 51 | 1 (2) |

* The starting number for this group was deliberately high to increase confidence in the results obtained with a single dose of 1·5 mg. This dose of the previous batch of MNU, sample A, had had "no effect" in our earlier experiments.

with a given fraction size took place (*e.g.* 0·1 mg MNU; experiments 4a, b, c, d, and e), after the appropriate number of doses had been administered animals not destined for further treatment were again randomly selected.

*Histology and electron microscopy.*—Animals were killed by cervical dislocation and examined for the presence of tumours. The urinary bladder was exposed, emptied by gentle pressure and, after clamping the urethra, cacodylate-buffered 4% formaldehyde (pH 7·3) was injected to fill but not overdistend the bladder. The serosal surface was then bathed with the fixative, and after 4 min the bladder was excised, opened and inspected for macroscopic abnormalities (*e.g.*, thickened areas, tumours and calculi). Representative samples were either further fixed in formalin for light microscopy or cut into 1mm$^3$ blocks and post-fixed in cold cacodylate-buffered 1% osmium tetroxide for electron microscopy. Other organs were examined for gross abnormalities, and the kidneys, lungs, liver, uterus, spleen and pancreas routinely processed for histology. All specimens for histology were fixed in formalin, embedded in paraffin wax, sectioned, and stained with haematoxylin and eosin. Thin sections ($\sim$80 nm) of Spurr-embedded bladder were contrast-stained with uranyl acetate and lead citrate for electron microscopy, and semi-thin (1$\mu$m) sections stained with toluidine blue for high-resolution light microscopy. At least 3 blocks from each bladder were examined by the latter method to complement the results obtained by conventional histology. The few animals that were found dead were processed for histology only.

### RESULTS

### *Tumour incidence after single graded doses of MNU*

The incidence of bladder tumours observed after single graded doses of MNU is plotted in Fig. 1. Two control

FIG. 1.—Rat urinary bladder tumour incidence in response to single graded doses of MNU. (Two separate control groups are shown for the zero dose.)

FIG. 2.—Bladder tumour incidence in response to multiple doses (at 2-week intervals) of 0·1 (■) 0·5 (●) and 1·5 (▲) mg MNU. (Open symbols show results from experiments with an interval of 25 weeks between the doses.)

groups were untreated. It can be seen that as the amount of MNU administered was increased, there was a progressive rise in tumour incidence. However, even at the highest dose, tumour incidence remained low (19%). On the basis of these data, a bladder-tumour incidence of 100% appears to be unobtainable from a single sub-lethal dose of this carcinogen.

*Tumour incidence after multiple doses of MNU*

The effect of splitting a given total dose into a series of smaller fractions was investigated. Two, 3 and 4 consecutive doses of 0·1 mg MNU were administered to separate groups of animals. Corresponding experiments were run using repeated doses of 0·5 and 1·5 mg MNU. The results (Fig. 2) show a clear relationship between tumour incidence and the number of doses in each case. For any given number of doses, the tumour yield rises as the dose level is increased, and with 3 doses of 1·5 mg tumour incidence is 100%. Expressing the same results in terms of the cumulative amount of MNU administered (Fig. 3), it can be seen that for any given total dose of MNU, tumour incidence was greater after smaller fractions of MNU than after larger fractions.

FIG. 3.—Same data as in Fig. 2, plotted against cumulative dose of MNU. (The symbol × represents tumour incidence after a single dose of 1·0 mg MNU.)

*Effect of interval between fractions*

The experimental groups given 2 doses of 0·1 and 0·5 mg MNU 2 weeks apart were compared with corresponding groups in which the same doses were separated by

FIG. 4.—Comparison of incidences of hyperplasias and tumours after single graded doses of MNU (A) and multiple doses of 0·1 mg MNU (B).

25 weeks. This extended interval did not reduce the tumour incidence (Fig. 2).

*Incidence of hyperplasia*

MNU-treated bladders showed a much higher incidence of hyperplasia than the controls (Fig. 4). Though the response was dose-related, the increase in hyperplasia with dose was less marked with single graded doses (Fig. 4A) owing to the high incidence even at the lowest dose (0·1 mg).

*Presence of calculi*

Thirty-two per cent of tumour-bearing bladders contained calculi or showed calcification, but this incidence was independent of MNU dose. Calculi were not found in the absence of a tumour in the present series of experiments. They varied in form and number, 2 extreme examples being illustrated in Fig. 5.

*Histopathology*

*Short-term effects.*—To examine the short-term effects of MNU treatment on the bladder, groups of rats separate from the main series of experiments were given single doses of 0·1, 0·2, 0·3, 0·4 and 0·5 mg MNU. Even at the lowest dose, toxic damage (notably intracellular oedema and stripping of superficial cells) was evident at 1–3 days after treatment, and mild focal hyperplasia up to 3 weeks. A progressively more marked response was

FIG. 5.—Calculi from tumour-bearing bladders. A, single calculus; B, multiple calculi from a single bladder (treatment; 2 × 0·5 mg MNU with a 25-week interval).

6
7
8
9
10
11

found as the dose increased, though even at 0·5 mg the effects remained mild and focal, and were detected only after thorough searching.

*Terminal findings.*—Although the incidence of focal and/or widespread urothelial hyperplasia and neoplasia depended on the number of doses and quantity of MNU administered, the principal pathological features were common to all the experimental groups. Most tumours were of the transitional-cell type, though 5% were of connective-tissue origin. Exceptionally, both carcinoma and sarcoma were present in the same bladder.

Some animals in the groups receiving single doses or multiple low doses of MNU retained a normal pattern of urothelial differentiation (Fig. 6). In the 0·1mg series, for example, the proportion of bladders with only normal-looking urothelium ranged from 53% in rats receiving a single dose down to 14% in those treated with 4 doses. A reduction in the number of bladders with normal differentiation was also found as single graded levels were increased (*e.g.*. 0·5 mg MNU, 42% normal; 1·0 mg MNU, 26% normal). It is noteworthy that although most control bladders showed normal urothelial differentiation, 10% had mild focal hyperplasia, and 1 tumour was found at the end of the 2-year period.

Hyperplasias were categorized as simple, papillary or nodular, though gradations between these forms and combinations of them were common in all the treated groups. Simple hyperplasias were characterized by the presence of a thickened urothelium in which an orderly differentiation from basal to superficial cell layers was apparent. Superficial cells were sometimes flattened, though not necessarily fully differentiated, and blood vessels were often conspicuous at the base of the urothelium. As the thickness of the urothelium increased, there was a loss of cellular organization, and blood vessels were frequently found growing up into or arching within the urothelium. Some hyperplasias displayed a distinct nodular growth pattern at an early stage (Fig. 7). Further development of the nodules was associated with increasing cellular pleomorphism and infiltration of urothelial cells into the stroma (Fig. 8). Lesions at this stage were classified as transitional-cell carcinoma with a nodular growth pattern.

Papillary hyperplasias were more common than the nodular variety, and were associated with an exophytic proliferation of blood vessels towards the bladder lumen. Even in mildly hyperplastic bladders, early blood-vessel development and marked atypia and disorganization in the neighbouring urothelial cells were sometimes seen (Fig. 9). It is a matter of judgement whether the degree of cell atypia is sufficient to classify this type of lesion as carcinoma *in situ*. Such "early" lesions were frequently associated with well developed papillary tumours, like those illustrated in Figs. 10 and 11. Fig. 10 shows part of an extensive early papillary tumour, and Fig. 11 a discrete localized one. In the latter, invasion has taken place into the supporting stalk of

FIG. 6.—Urothelium showing normal differentiation into basal, intermediate and superficial cell layers from an animal killed 2 years after receiving 3 × 0·1 mg MNU. No abnormality was detected elsewhere in this bladder. Toluidine blue-stained semi-thin section. × 305.

FIG. 7.—Early nodular hyperplasia associated with sub-urothelial lymphocytic infiltration in an animal treated with 4 × 0·1 mg MNU. Toluidine blue. × 170.

FIG. 8.—Part of a tumour with a nodular growth pattern from an animal treated with 2 × 0·5 mg MNU (25 weeks between doses). H. & E., wax section. × 110.

FIG. 9.—Mildly hyperplastic urothelium showing focal areas of atypical cells in which there is a disorientated and differential growth pattern. (Treatment: 4 × 0·1 mg MNU.) Toluidine blue. × 235.

FIG. 10.—Part of an extensive early papillary tumour from an animal that received 4 × 0·1 mg MNU. Toluidine blue. × 170.

FIG. 11.—Small discrete papillary tumour from an animal treated with 2 × 0·5 mg MNU. Nests of invasive cells penetrate into the tumour stalk. The surrounding urothelium is moderately hyperplastic. Toluidine blue. × 75.

the tumour, but not below the level of the surrounding hyperplastic urothelium.

In more advanced papillary tumours, nests of urothelial cells were found in the stroma below the tumour stalk (Fig. 12), sometimes extending deep into the bladder wall (Fig. 13). The invading cells often appeared increasingly pleomorphic, bearing little resemblance to the urothelial cells from which they were derived (Fig. 14). Most of the papillary lesions, whether or not there were invasive regions, were well-differentiated transitional-cell tumours and often showed areas of squamous metaplasia (Fig. 15).

Cystitis cystica and adenocarcinomas were not observed in these animals.

No metastases from the bladder tumours were found in other organs, though a few neoplasms were found in the uterus and the kidneys of some of the MNU treated animals.

A single untreated control rat developed a bladder tumour, detected at 86 weeks after the start of the experiment. This animal also had an extensive papillary tumour of the kidney calyx, associated with a large kidney stone. Calculi were also present in the bladder, but otherwise no macroscopic abnormality was evident. On microscopic examination, however, a flat invasive transitional-cell tumour (P2) which displayed focal regions of mucous metaplasia was found. The surface urothelium was of almost normal thickness but invaginated as projections into the stroma (Fig. 16). This growth pattern was rarely found after MNU treatment.

*Electron microscopy*

A scalloped luminal-membrane profile of semi-rigid concave plaque regions separated by flexible interplaque peaks is the hallmark of normal differentiation in the urothelium, and was present in most of the untreated control animals. Such a structure was also typical of the carcinogen-treated bladders diagnosed as normal by light microscopy, and was also seen in some regions of normal appearance from tumour-bearing bladders (Fig. 17).

Transitional-cell tumours displayed 2 characteristic structural features—pleomorphic microvilli and a prominent glycocalyx—at the luminal face of some but not all surface cells. Often these structures were associated with one another, though this was not invariable. The tumour-cell surface in Fig. 18, for example, has abundant well-developed microvilli but no glycocalyx, whereas the irregular but non-microvillous surface depicted in Fig. 19 shows a conspicuous glycocalyx. Considerable morphological variation in both the microvilli and the glycocalyx was found, though the exact form of structural differentiation tended to remain constant over the surface of a given cell. Some microvilli consisted of rather short, stubby projections with knobby heads (Fig. 20) and apparently developed from interplaque regions of the luminal membrane, as normal differentiation was lost. Glycocalyx material was more profuse over the surface of microvilli than on the intervening membrane regions, though it was rarely completely excluded from them (Figs. 20 and 21). The filaments comprising the glycocalyx ranged from coarse electron-dense beaded structures to fine ones (Figs 19–22). Longer filaments were often branched, and although the branches

---

FIG. 12.—Papillary tumour with early stomal invasion. (2 × 0·5 mg MNU; 25 weeks between doses.) H. & E. × 160.

FIG. 13.—Invasive cords and nests of atypical urothelial cells deep in the bladder wall beneath an advanced papillary tumour. (2 × 0·5 mg MNU with 25-week interval.) H. & E. × 100.

FIG. 14.—Detail of pleomorphic urothelial cells at invasive base of a tumour in an animal treated with a single dose of 1·0 mg MNU. Toluidine blue. × 190.

FIG. 15.—Squamous metaplasia and keratinization in part of a papillary tumour. (2 × 0·5 mg MNU with 25-week interval.) Toluidine blue. × 145.

FIG. 16.—Surface region from part of the flat invasive urothelial tumour found in a single control animal. H. & E. × 155.

17
18
19
20
21
22

usually remained of constant thickness, sometimes a progressive reduction in the diameter of each new branch was apparent (Figs 20 and 21).

The single bladder tumour from the control group also revealed short microvilli at the luminal surface which were covered with a fine filamentous glycocalyx (Fig. 22).

### DISCUSSION

MNU has a short (5–10 min) half-life in the body (Swann, 1968), but its rate of decay is pH-dependent, and at the normal pH of rat urine (6·0–6·5; Chowaniec & Hicks, 1979) it will persist much longer. Using tritiated MNU and high-pressure liquid chromatography, we had previously demonstrated the half-life of MNU in Hanks' balanced salt solution to be ~300 min at pH 6·0, 150 min at pH 6·3 and 90 min at pH 6·5 (Knowles, Moore and Hicks, umpublished). The MNU solution instilled into the bladder in the current experiments mixes with fresh urine (pH 6·0–6·5) arriving from the kidneys, and clearly persists long enough to react with macromolecules in the urothelial cell. Indeed, using our instillation technique, Cox & Irving (1977) demonstrated temporary alkylation of various bases in rat urothelial cell DNA, and the persistence of $O^6$-methylated guanine with accumulation of the $O^6$ product after repeated instillations of MNU into the bladder. Provided the method of preparation and instillation of the MNU solution is carefully standardized, our results demonstrate that any pH-related decay does not confound the dose-related response of the urothelium to MNU.

As can be seen from Fig. 1, there is a good dose-related response of the urinary bladder to the carcinogenic effect of MNU administered in single graded doses. Furthermore it can be seen from Tables I & II and Fig. 2 that the effect of repeated small doses of the carcinogen is cumulative and that, using different aliquots, there is a dose–response curve. When the tumour incidence is plotted against the total cumulative dose (Fig. 3) it can be seen that for any given total dose the tumour response is greater if the MNU had been administered in the smaller fractions. This reflects the dose-related toxicity of MNU. Larger fractions may transform more cells, but at the same time fewer cells survive toxic damage, so that the surviving population available for reaction with subsequent doses is progressively reduced.

Although much higher doses of the first batch of MNU (sample A) had to be used to obtain the same effect, a good dose-related response had been obtained with that preparation also (Hicks *et al.*, 1978). With sample A, 1·5 mg was sub-carcinogenic, whereas 1·5 mg of later freshly prepared samples of MNU (sample B) produced a bladder cancer incidence of ~20%. Nevertheless, the results obtained with sample A remain valid, since there were always appropriate concurrent controls, and the dose response remained consistent for several years.

Fig. 17.—Thin-section electron micrograph showing the luminal face of an area of normal-looking urothelium from a tumour-bearing bladder. The scalloped profile is identical to that seen in untreated animals. (2 x 0·5 mg MNU; 25-week interval.) x 46,800.

Fig. 18.—EM of a tumour-cell surface from the same bladder as in Fig. 17. Long microvilli (in cross-section) are abundant but no glycocalyx is present. x 25,200.

Fig. 19.—Elsewhere in the same tumour microvilli are absent at the surface but a prominent beaded glycocalyx is visible. x 50,400.

Fig. 20.—This example further illustrates the variety of structural differentiation within the same tumour. Here a fronded glycocalyx covers short microvilli, but is absent from the intervening membrane. x 41,400.

Fig. 21.—Example of a beaded glycocalyx on short surface projections. (Tumour from animal treated with 2 x 0·5 mg MNU; 25-week interval.) x 47,700.

Fig. 22.—Fine filamentous glycocalyx at the surface of the single control bladder tumour. x 75,600.

Similar variability between different batches of another carcinogen, N-(4-(5-nitro-2-furyl)-2-thiazolyl) formamide (FANFT) has been reported. In some experiments, 6 weeks' feeding of 0·2% dietary FANFT proved to be a sub-threshold dose (Jacobs *et al.*, 1977) whereas in others it produced a 20% bladder-tumour incidence (Cohen *et al.*, 1979). Again since concurrent controls were used the results are valid for both sets of experiments.

It was hoped that an absolute dose of MNU (sample B) suitable for initiation in multistage carcinogenesis could be derived from the data in Figs 1 & 2. Ideally, such a dose should be high enough to initiate a large proportion of cells, but too low to complete the subsequent promotion and propagation stages of carcinogenesis. In that situation, no tumours would developed until the initiated cells were acted upon by promoters or further doses of a carcinogen. However, inspection of Fig. 1 shows that there is no threshold dose of sample B below which tumours were completely absent. Furthermore, one animal in a group of 51 controls also develop a bladder tumour. This is the only "spontaneous" bladder tumour observed in our Wistar rat colony during 15 years, and in this instance may well have been seeded from the transitional-cell tumour found higher up in the urinary tract in the renal pelvis. These findings emphasize the difficulties in achieving the ideal characteristics for a multistage-carcinogenesis animal model *in vivo*. In practice, determination of a true initiating dose may be unrealistic. A compromise has to be made based on the minimum tumour incidence induced by a single dose that is consistent with a markedly increased incidence on subsequent dosing. On this basis, the present results suggest a single treatment of 0·3–0·5 mg MNU as a suitable threshold dose for studying multistage carcinogenesis in the Wistar rat model. It must be remembered, however, that the persistence (and therefore the effective dose) of MNU in the bladder will be affected by urinary pH, and that this in turn may be altered by diet. Furthermore, different mouse strains are known to have different susceptibilities to individual carcinogens (Andervont & Edgcomb, 1956; Festing, 1975) and the same may well be true for different rat strains. It may thus prove necessary to adjust the threshold dose used in any particular laboratory and for any particular strain of rat. In such experiments, the requirement for concurrent controls using the same batch of carcinogen cannot be emphasized too strongly.

In the current series of experiments, tumour incidence remained essentially unchanged on extending the interval between 2 doses of MNU, confirming that the carcinogenic damage induced by the first dose persists over a long period. This is consistent both with the original concept of initiation as a permanent alteration to the cell (Berenblum, 1941) and with the reported persistence of $O^6$-methyl-guanine in the DNA of MNU-treated urothelium (Cox & Irving, 1977). However, a reduced bladder-tumour incidence was observed in another system in which a delay was interposed between initiation and promotion treatments. In the FANFT model, when saccharin was used as a promoter, fewer tumours were found when 6 weeks were allowed between the FANFT and saccharin treatments than when no interval was given (Cohen *et al.*, 1979). In the same model, Arai *et al.* (1977) demonstrated the promoting activity of cyclophosphamide, but this activity disappeared if administration was delayed for 6 weeks after completion of the FANFT treatment. Thus, in contrast to the carcinogenic damage induced by MNU, some of the damage induced by FANFT may be repaired.

The mild focal hyperplasia in 10% of untreated bladders at the end of the 2-year period indicates that the experimental results should be interpreted against a background of age-related pathological change. A few hyperplasias in control Wistar males (but not females) were recorded in previous experiments from this laboratory (Chowaniec & Hicks, 1979).

Our finding that the incidence of hyperplasias as well as tumours is dose-related (Fig. 4) supports the histopathological evidence that tumours develop from hyperplastic lesions. However, not all hyperplasias necessarily progress to tumours, and the exact significance of hyperplasia in the biogenesis of tumours remains under discussion. A "precursor-product" relationship between *persistent* hyperplasias (*i.e.* those persisting 9 months or longer) and tumours which had developed 9 months later, was established in 2-acetylaminofluorene (2-AAF)-treated BALB/c mice, even though some early hyperplasias in that system regress if treatment is stopped (Littlefield *et al.*, 1979). The early hyperplasia (*i.e.* at 2 days to 3 weeks) after a single dose of MNU or a single dose of cyclophosphamide rapidly regresses, and normal differentiation is subsequently restored (Hicks & Wakefield, 1972; Koss & Lavin, 1970). Although a few of the hyperplasias present at the end of the current experiments could possibly have arisen as a late temporary response (*e.g.* to the irritant effect of a calculus) it seems probable that most were of the persistent type and, as in the 2-AAF system, represent preneoplastic lesions potentially leading to tumours. However, within the time-scale of the experiments, clearly only a small proportion of these hyperplasias is able to progress to tumours, as is shown by Fig. 4.

The irritant effect of calculi and the consequent increase in cell turnover is thought to give a propagating stimulus to tumour growth in the rodent bladder (Chapman *et al.*, 1973) and the presence of calculi in one-third of the tumour-bearing bladders in the present experiments is consistent with this view. However, since the presence of calculi was not related to MNU dose, and not only do some tumours develop in the absence of calculi but also calculi sometimes occur in tumour-free bladders (Hicks & Chowaniec, 1978), the presence of a calculus cannot be considered as obligatory to tumour formation (*cf.* Clayson, 1974).

The hyperplastic and neoplastic lesions of the urothelium induced by MNU appear similar to those induced by other bladder carcinogens (*e.g.* FANFT, BBN and bracken fern), and the pathways of tumour development are probably common to all these experimental systems (Kunze *et al.*, 1976; Tiltman & Friedell, 1971; Pamukcu *et al.*, 1976; Hicks & Chowaniec, 1978). In previous ultrastructural investigations, pleomorphic microvilli and a filamentous or beaded glycocalyx have been identified as morphological markers of neoplastic transformation in the bladder (Hicks & Wakefield, 1976; Newman & Hicks, 1977; Shirai *et al.*, 1978). Our present studies demonstrate that these structures may exhibit a striking variety of form, and that they occurred in both the one "spontaneous" and the MNU-induced rat bladder tumours. Although pleomorphic microvilli have been consistently regarded as a characteristic marker for neoplastic transformation in the urothelium (Newman & Hicks, 1977; Jacobs *et al.*, 1977; Shirai *et al.*, 1978) a recent report describes their occasional presence in severe reversible hyperplasia (Fukushima *et al.*, 1981). An occasional cell with microvilli is sometimes found in urine sediments from healthy humans with no neoplastic disease of the urinary tract, but such cells are far more numerous in sediments from bladder-cancer patients (Newman & Hicks, 1981). Both qualitative and quantitative changes are thus associated with neoplastic transformation and, rather than placing absolute reliance on any single phenotypic marker, both must be assessed when judging the probability of the urothelium progressing to neoplasia.

In conclusion, the present results with fractionated doses of MNU, together with previous reports from this laboratory (Hicks & Chowaniec, 1977; Hicks *et al.*, 1978; Hicks, 1980), demonstrate a multi-step process of carcinogenesis in the bladder. This suggests that bladder carcinogenesis has many characteristics in common with the classical multistage model derived from studies on the mouse epidermis (Berenblum, 1974). Evidence has accumulated that the multistage model is applicable to carcinogenesis in the liver (Peraino *et al.*, 1973; Farber & Solt, 1978; Pitot *et al.*, 1978) and possibly in the colon (Reddy *et al.*, 1978) and lung (Witschi & Lock, 1978). Furthermore, epidemiological studies suggest that the development of many human carcinomas also follows a temporal sequence of events (Peto, 1977). The multistage model thus provides a useful framework within which to analyse the development of neoplasia in epithelial tissues.

This work was supported by a generous grant from the Cancer Research Campaign of Great Britain. We thank Miss Leonora Simon and Mr Roger Merrick for technical assistance and Miss Ruth Fitch for typing the manuscript.

### REFERENCES

ANDERVONT, H. B. & EDGCOMB, J. H. (1956) Response of seven inbred strains of mice to percutaneous applications of 3-methylcholanthrene. *J. Natl Cancer Inst.*, **17**, 481.

ARAI, M., COHEN, S. M. & FRIEDELL, G. H. (1977) Promoting effect of cyclophosphamide (CP) on rat urinary bladder carcinogenesis following initiation by N-(-4-(5-nitro-2-furyl)-2-thiazolyl)-formamide (FANFT). *Proc. Jap. Cancer Assoc.*, Tokyo: Japanese Cancer Association. p. 39.

BERENBLUM, I. (1941) The mechanisms of carcinogenesis: A study of the significance of cocarcinogenic action and related phenomena. *Cancer Res.*, **1**, 807.

BERENBLUM, I. (1974) *Carcinogenesis as a Biological Problem. Frontiers of Biology*, Vol. 34. (Eds Neuberger & Tatum.) Amsterdam: North-Holland. p. 1.

CHAPMAN, W. H., KIRCHHEIM, D. & MCROBERTS, J. W. (1973) Effect of urine and calculus formation on the incidence of bladder tumours in rats implanted with paraffin wax pellets. *Cancer Res.*, **33**, 1225.

CHOWANIEC, J. & HICKS, R. M. (1979) Response of the rat to saccharin with particular reference to the urinary bladder. *Br. J. Cancer*, **39**, 355.

CLAYSON, D. B. (1974) Bladder cancer in rats and mice: Possibility of artifacts. *J. Natl Cancer Inst.*, **52**, 1685.

COHEN, S. M., ARAI, M., JACOBS, J. B. & FRIEDELL, G. H. (1979) Promoting effect of saccharin and DL-tryptophan in urinary bladder carcinogenesis. *Cancer Res.*, **39**, 1207.

COX, R. & IRVING, C. C. (1976) Effect of *N*-methyl-*N*-nitrosourea on the DNA of rat bladder epithelium. *Cancer Res.*, **36**, 4114.

COX, R. & IRVING, C. C. (1977) Selective accumulation of $O^6$-methylguanine in DNA of rat bladder epithelium after intravesical administration of *N*-methyl-*N*-nitrosourea. *Cancer Lett.*, **3**, 265.

FARBER, E. & SOLT, D. (1978) A new liver model for the study of promotion In *Carcinogenesis, Vol. 2. Mechanisms of Tumour Promotion and Cocarcinogenesis*, (Eds Slaga *et al.*) New York: Raven Press, p. 443.

FESTING, M. F. W. (1975) A case for using inbred strains of laboratory animals in evaluating the safety of drugs. *Fd. Cosmet. Toxicol.*, **13**, 369.

FREI, J. V. & LAWLEY, P. D. (1975) Methylation of DNA in various organs of C57BL mice by a carcinogenic dose of *N*-methyl-*N*-nitrosourea and stability of some methylation products up to 18 hours. *Chem. Biol. Interact.*, **10**, 413.

FUKUSHIMA, S., COHEN, S. M., ARAI, M., JACOBS, J. B. & FRIEDELL, G. H. (1981) Scanning electron microscopic examination of reversible hyperplasia of the rat urinary bladder. *Am. J. Pathol.*, **102**, 373.

HICKS, R. M. (1980) Multistage carcinogenesis in the urinary bladder. *Br. Med. Bull.*, **36**, 39.

HICKS, R. M. & CHOWANIEC, J. (1977) The importance of synergy between weak carcinogens in the induction of bladder cancer in experimental animals and humans. *Cancer Res.*, **37**, 2943.

HICKS, R. M. & CHOWANIEC, J. (1978) Experimental induction, histology and ultrastructure of hyperplasia and neoplasia of the urinary bladder epithelium. *Int. Rev. Exp. Pathol.* **18**, 199.

HICKS, R. M., CHOWANIEC, J. & WAKEFIELD, J. ST J. (1978) The experimental induction of bladder tumours by a two-stage system. In *Carcinogenesis, Vol. 2. Mechanisms of Tumor Promotion and Cocarcinogenesis*, (Eds Slaga *et al.*) New York: Raven Press. p. 475.

HICKS, R. M. & WAKEFIELD, J. ST J. (1972) Rapid induction of bladder cancer in rats with *N*-methyl-*N*-nitrosourea. I. Histology. *Chem. Biol. Interact.*, **5**, 139.

HICKS, R. M. & WAKEFIELD, J. ST J. (1976) Membrane changes during urothelial hyperplasia and neoplasia. *Cancer Res.*, **36**, 2502.

HICKS, R. M., WAKEFIELD, J. ST J. & CHOWANIEC, J. (1975) Evaluation of a new model to detect bladder carcinogens or co-carcinogens: Results obtained with saccharin, cyclamate and cyclophosphamide. *Chem. Biol. Interact.*, **11**, 225.

HOOSON, J., HICKS, R. M., GRASSO, P. & CHOWANIEC, J. (1980) Ortho-toluene sulphonamide and saccharin in the promotion of bladder cancer in the rat. *Br. J. Cancer*, **42**, 129.

JACOBS, J. B., ARAI, M., COHEN, S. M. & FRIEDELL, G. H. (1977) A long term study of reversible and progressive urinary bladder cancer lesions in rats fed *N*-(4-(5-nitro-2-furyl)-2-thiazolyl)formamide. *Cancer Res.*, **37**, 2817.

KOSS, L. G. & LAVIN, P. (1970) Effects of a single dose of cyclophosphamide on various organs in the rat. II. Response of urinary bladder epithelium according to strain and sex. *J. Natl Cancer Inst.*, **44**, 1195.

KUNZE, E., SCHAUER, A. & SCHATT, S. (1976) Stages of transformation in the development of *N*-butyl-*N*-(4-hydroxybutyl)-nitrosamine-induced transitional cell carcinomas in the urinary bladder of rats. *Z. Krebsforsch.*, **87**, 139.

LITTLEFIELD, N. A., GREENMAN, D. L., FARMER,

J. H. & SHELDON, W. G. (1979) Effects of continuous exposure to 2-AAF on urinary bladder hyperplasia and neoplasia. *J. Environ. Pathol. Toxicol.*, **3**, 35.

MOHR, U., GREEN, U., ALTHOFF, J. & SCHNEIDER, P. (1979) Syncarcinogenic action of saccharin and sodium cyclamate in the induction of bladder tumours in MNU-pretreated rats. In *Health and Sugar Substitutes*, (Ed. Guggenheim.) Basle: Karger. p. 64.

NEWMAN, J. & HICKS, R. M. (1977) Detection of neoplastic and preneoplastic urothelia by combined scanning and transmission electron microscopy of urinary surface of human and rat bladders. *Histopathology*, **1**, 125.

NEWMAN, J. & HICKS, R. M. (1981) Diffuse neoplastic change in urothelium from tumour-bearing lower urinary tract. In *Scanning Electron Microscopy* Vol. III. (Ed. Johari) Chicago: SEM Inc. p. 1.

PAMUKCU, A. M., ERTÜRK, E., YALÇINER, S. & BRYAN, G. T. (1976) Histogenesis of urinary bladder cancer induced in rats by bracken fern. *Invest. Urol.*, **14**, 213.

PERAINO, C., FRY, J. M., STAFFELOT, E. & KISIELESKI, W. E. (1973) Effect of varying the exposure to phenobarbital on its enhancement of 2-acetyl-amino-fluorene-induced hepatic tumorigenesis in the rat. *Cancer Res.*, **33**, 2701.

PETO, R. (1977) Epidemiology, multistage models and short-term mutagenicity tests. In *Origins of Human Cancer, Book C, Human Risk Assessment*, Vol. 4. (Eds Hiatt *et al.*) New York: Cold Spring Harbor. p. 1403.

PITOT, H. C., BARSNESS, L. & KITAGAWA, T. (1978) Stages in the process of hepatocarcinogenesis in rat liver. In *Carcinogenesis, Vol. 2. Mechanisms of Tumor and Cocarcinogenesis.* (Eds Slaga *et al.*) New York: Raven Press. p. 433.

REDDY, B. S., WEISBURGER, J. H. & WYNDER, E. L. (1978) Colon cancer: Bile salts as tumour promotors. In *Carcinogenesis, Vol. 2. Mechanisms of Tumor Promotion and Cocarcinogenesis.* (Eds Slaga *et al.*) New York: Raven Press. p. 453.

SHIRAI, T., COHEN, S. M., FUKUSHIMA, S., HANANOUCHI, M. & ITO, N. (1978) Reversible papillary hyperplasia of the rat urinary bladder. *Am. J. Pathol.*, **91**, 33.

SWANN, P. F. (1968) The rate of breakdown of methylmethane sulphonate, dimethylsulphate and *N*-methyl-*N*-nitrosourea in the rat. *Biochem. J.*, **110**, 49.

TILTMAN, A. J. & FRIEDELL, G. H. (1971) The histogenesis of experimental bladder cancer. *Invest. Urol.*, **9**, 218.

WERNER, E. A. (1919) The constitution of the carbamides. IX. The interaction of nitrous acid and monosubstituted ureas. The preparation of diazomethane, diazoethane, diazo-n-butane and diazoisopentane. *J. Chem. Soc.*, **115**, 1093.

WITSCHI, H. & LOCK, S. (1978) Butylated hydroxytoluene: A possible promoter of adenoma formation in mouse lung. In *Carcinogenesis, Vol. 2. Mechanisms of Tumor Promotion and Cocarcinogenesis*, (Eds Slaga *et al.*) New York: Raven Press. p. 465.